# Low-intensity shockwave therapy in men’s health: Insights from a systematic review on erectile dysfunction and CP/CPPS in the Middle East

**DOI:** 10.1080/20905998.2025.2611547

**Published:** 2026-01-05

**Authors:** Ahmad Majzoub, Mohamed Arafa, Osama Shaeer, Ahmed Loghmari, Amr Abdelhamed, Mohamed Wael Ragab, Haitham Elbardisi

**Affiliations:** a Department of Urology, Hamad Medical Corporation, Doha, Qatar; b Department of Clinical Urology, Weill Cornell Medicine-Qatar, Doha, Qatar; c Andrology Department, Cairo University, Cairo, Egypt; d Department of Urology, College of Medicine, Qatar University, Doha, Qatar; e Urology Department, Sahloul Teaching Hospital, University of Sousse, Sousse, Tunisia; f Faculty of Medicine, University of Sousse, Sousse, Tunisia; g Dermatology, Venereology & Andrology Department, Faculty of Medicine, Sohag University, Sohag, Egypt

**Keywords:** Low-intensity shockwave therapy, erectile dysfunction, chronic pelvic pain syndrome, Middle East, men’s health, non-invasive therapy

## Abstract

Low-intensity shockwave therapy (LiSWT) has gained attention as a non-invasive treatment for various men’s health conditions, including erectile dysfunction (ED), Peyronie’s disease (PD), and chronic prostatitis/chronic pelvic pain syndrome (CP/CPPS). However, despite its increasing use, robust evidence supporting its effectiveness remains limited. This systematic review aims to evaluate the efficacy and safety of LiSWT in managing men’s health conditions within the Middle East to provide region-tailored clinical recommendations. A systematic search was conducted using PubMed to identify studies that evaluated LiSWT in ED, PD, or CP/CPPS. Studies were included if they employed a prospective design and reported outcomes based on validated questionnaires. The quality of included studies was assessed using the Cochrane Risk of Bias Tool and the Newcastle-Ottawa Scale. Fourteen studies met the inclusion criteria; 7 RCTs, 2 non-randomized comparative studies, and 5 observational studies. Ten studies focused on ED, while 4 evaluated CP/CPPS. No studies on PD were identified. For ED, LiSWT demonstrated significant improvements in IIEF-5 and EHS scores across most studies, with better outcomes observed in younger patients, those with milder ED, and shorter disease duration. However, some studies found no significant differences between LiSWT and PDE-5 inhibitors or sham treatments, with benefits often diminishing over time. For CP/CPPS, LiSWT showed significant reductions in NIH-CPSI and IPSS scores, with improvements sustained up to 12 months. Study quality ranged from moderate to high, though limitations such as high risk of bias in some RCTs and selection bias in observational studies were noted. While LiSWT represents a promising therapeutic option for ED and CP/CPPS, its application should be costumized to meet the specific needs of individual patients. Future research should focus on developing standardized protocols and conducting large-scale randomized controlled trials to establish its long-term efficacy and optimize clinical use in the Middle East.

## Introduction

Low-intensity shockwave therapy (LiSWT) is a non-invasive therapeutic modality that has been applied across various medical fields due to its unique mechanisms of action. By delivering focused acoustic waves to targeted tissues, LiSWT is believed to promote angiogenesis, enhance blood flow, and stimulate tissue regeneration, making it a promising option for conditions involving vascular or inflammatory pathophysiologies [1,2]. Initially utilized in orthopedics for tendon and bone healing [3,4], LiSWT has since been applied to cardiovascular diseases [5], wound healing [6], and even certain neurological conditions [7,8]. Its versatility stems from its ability to induce cellular repair and regeneration, offering significant therapeutic potential across diverse clinical applications.

In men’s health, LiSWT has gained recognition as an innovative treatment for erectile dysfunction (ED), chronic prostatitis/chronic pelvic pain syndrome (CP/CPPS), and Peyronie’s disease (PD) [9–11]. Its application was first explored in PD and the earliest study demonstrated its efficacy in reducing the pain associated with the condition rather than addressing the deformity [12]. Subsequently, LiSWT was expanded to CPPS for its anti-inflammatory and analgesic properties [13], and more recently, to ED, for its potential to improve penile hemodynamics and promote tissue remodeling [14]. Over the past two decades, numerous studies have evaluated LiSWT in these conditions, with the majority focusing on ED [9,15,16]. These studies have consistently highlighted its safety and tolerability. However, questions remain regarding its efficacy, particularly due to variability in study designs, treatment protocols, and outcome measures, which have resulted in a discrepancy between its widespread use and the available evidence supporting its effectiveness.

International guidelines reflect this uncertainty. The American Urological Association (AUA) classifies LiSWT as investigational for ED, citing inconsistent findings and limited sample sizes in randomized controlled trials (RCTs) [17]. The European Association of Urology (EAU) recommends LiSWT as an alternative therapy in specific scenarios, such as for patients with mild vasculogenic ED, those unwilling to use oral therapies, and PDE-5i poor responders [18]. Similarly, the European Society for Sexual Medicine (ESSM) highlights LiSWT’s safety but questions its efficacy for ED, while acknowledging potential benefits for CP/CPPS and pain relief in PD [19]. The Canadian Association of Urology (CAU) conditionally recommends against LiSWT for ED, underscoring the need for robust, long-term evidence to support its use [20].

In the Middle East, LiSWT’s role warrants particular attention. Cultural, financial, and healthcare barriers often limit access to more definitive treatments, such as penile prostheses, while the high prevalence of vasculogenic ED increases the demand for alternative therapies [21,22]. Moreover, excessive advertising of LiSWT in the private sector, often without adequate clinical evidence, emphasizes the need for region-specific studies to establish evidence-based guidelines.

This systematic review examines the application of LiSWT in the Middle East, with a focus on ED, PD, and CP/CPPS. By synthesizing findings from regional studies, we aim to provide insights into its utility, guide clinical decision-making, and support the development of tailored recommendations for the Middle Eastern population.

## Methods

### Search strategy

A systematic review was conducted following the Preferred Reporting Items for Systematic Reviews and Meta-Analyses (PRISMA) guidelines. The search was performed using PubMed as the primary database to identify studies evaluating the effects of LiSWT on men’s health conditions. The search strategy included relevant keywords and Medical Subject Headings (MeSH) terms such as ‘low-intensity shockwave therapy,’ ‘erectile dysfunction,’ ‘Peyronie’s disease,’ ‘chronic prostatitis,’ ‘chronic pelvic pain syndrome,’ and ‘Middle East.’ The details of the searched terms are presented in supplementary table S1. The search was restricted to studies conducted in or originating from countries within the Middle East and North Africa (MENA) region, and no language restrictions were applied.

### Inclusion and exclusion criteria

Studies were included if they met the following criteria: (1) Evaluated the use of LiSWT in patients with ED, PD, or CP/CPPS; (2) Conducted in or involved populations from the MENA region which include the 22 countries enrolled in the League of Arab States; (3) Employed a prospective study design, including randomized controlled trials (RCTs), non-randomized comparative studies, or observational studies; (4) Reported results based on measurable validated questionnaires.

Studies that were retrospective, reviews, or case reports with insufficient data or those focusing on LiSWT applications outside the scope of men’s health conditions (e.g. orthopedic use) were excluded.

### Study selection

Titles and abstracts of the identified articles were screened independently by two reviewers. Full-text articles were retrieved for studies that appeared to meet the inclusion criteria. Discrepancies were resolved through consensus or consultation with a third reviewer.

### Data extraction

Data were extracted using a standardized form and included: (1) Study design and population characteristics; (2) Intervention protocols (e.g. session frequency, energy intensity, shockwave device); (3) Outcome measures and follow-up duration; (4) Key findings and statistical significance.

### Quality assessment

The methodological quality of included studies was assessed using the Cochrane Risk of Bias Tool for RCTs [23] and the Newcastle-Ottawa Scale for non-randomized studies [24]. Discrepancies in quality assessment were resolved through discussion among the reviewers.

## Results

### Study selection and characteristics

The literature search identified 251 studies, of which 198 were excluded as they either were unrelated to LiSWT or explored its use in other medical conditions. An additional 39 studies were excluded for being reviews or case reports (Figure 1). Fourteen studies met the inclusion criteria, evaluating the impact of LiSWT on ED (*n* = 10) and CP/CPPS (*n* = 4) (Table 1). No studies evaluating the effect of LiSWT on PD were identified from the MENA region. All studies were prospective in design, including seven RCTs [25–31], two non-randomized comparative studies [32,33], and five observational studies [34–38].

**Figure 1.**
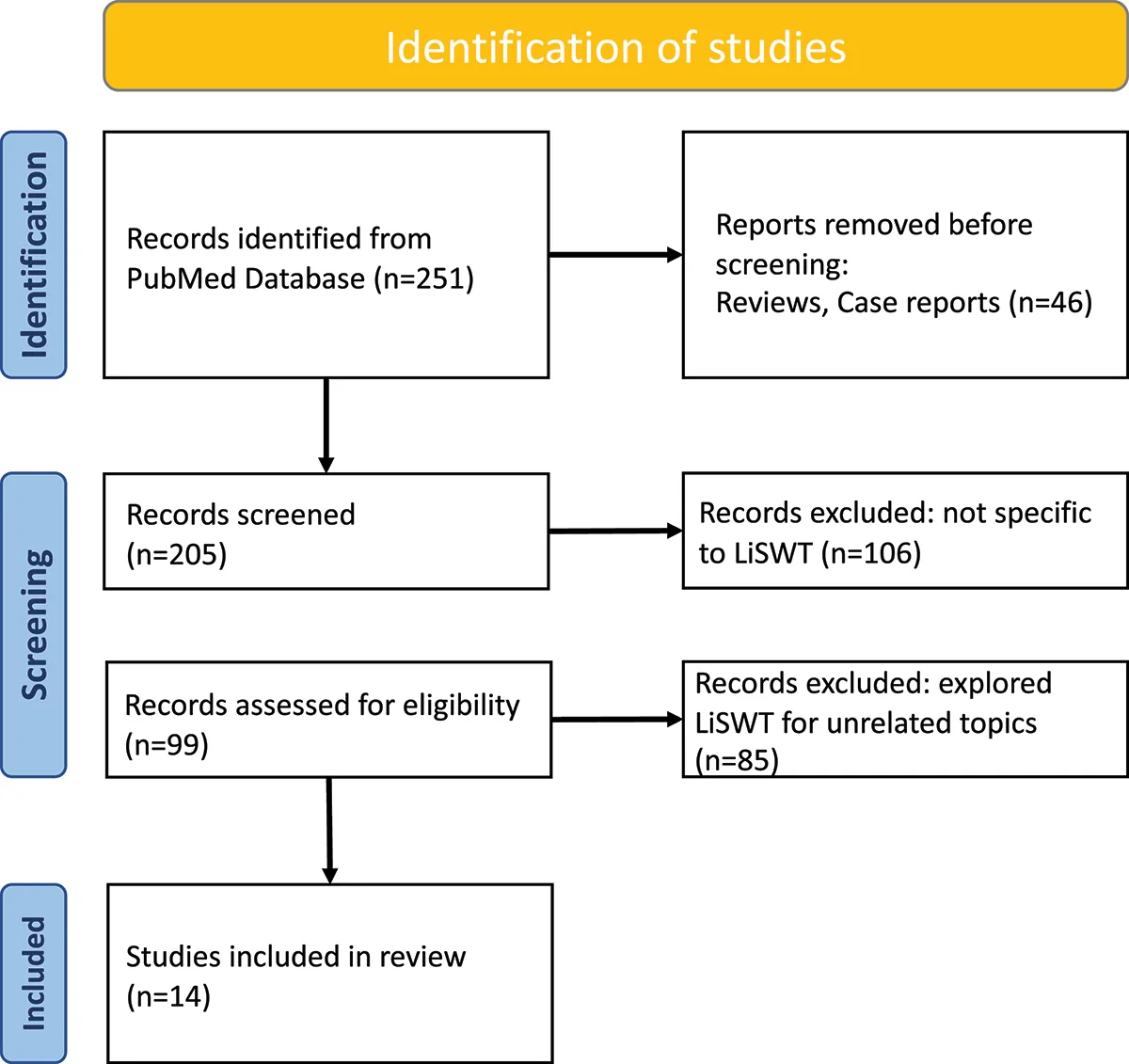
PRISMA flowchart of the systemic review.

**Table 1.** LiSWT protocol details for erectile dysfunction and chronic pelvic pain syndrome.

Study author	Design	Number of shocks	Device used	Frequency	Power (Energy density)	Duration
***Studies on erectile dysfunction***						
Abd El-Salam et al. 2024	Prospective	5000	NS	NS	0.09 mJ/mm^2^	Twice weekly for 3 weeks, repeated after 3-week rest.
Adeldaeim et al. 2020	Prospective	6000	PiezoWave2 (Richard Wolf)	8 Hz	0.16–0.20 mJ/mm^2^	6 sessions over 6 weeks.
Ismail et al. 2022	Prospective RCT	1800	Chattanooga Intelect RPW	120 shocks/min	0.09 mJ/mm^2^	2x/week for 3 weeks, then 3-week rest, then repeated.
Feghali 2023	Prospective	6000	PiezoWave2 (Richard Wolf)	8 Hz	0.16 mJ/mm^2^	4 or 6 weekly sessions depending on response.
Musa et al. 2020	Prospective	1500	Dornier Aries (Dornier MedTech)	NS	0.09 mJ/mm^2^	2x/week for 3 weeks, 3-week break, repeat until 12 sessions.
Salama et al. 2023	Prospective	3000	Shockmaster MP200 (Storz)	6 Hz	0.25 mJ/mm^2^	Twice weekly for 6 weeks.
Shakban et al. 2023	Prospective RCT	3600	Gymna Shock Master 300	120/min	0.09 mJ/mm^2^	Two sessions per week for 3 weeks.
Shendy et al. 2020	Prospective RCT	3000	Chattanooga Intellect Focus (CSW-400)	NS	0.09 mJ/mm^2^	Twice weekly for 3 weeks, repeat after 3 weeks.
Zanaty et al. 2022	Prospective RCT	6000	PiezoWave2 (Richard Wolf)	NS	NS	6 sessions (2/week).
Zewin et al. 2018	Prospective RCT	1500	Dornier Aries (Dornier MedTech)	120/min	0.09 mJ/mm^2^	12 sessions: 2/week for 3 weeks, 3-week break, repeat.
***Studies on chronic pelvic pain syndrome***						
Khalafalla et al. 2023	Prospective	3000	Duolith SD1 Ultra (Storz)	150/min	0.1 to 2.5 mJ/m^2^	4 weekly sessions.
Sakr et al. 2022	Prospective RCT	3000	Duolith SD1 Ultra (Storz)	4 Hz	0.25 mJ/mm^2^	4 weekly sessions.
Salama and Abouelnaga 2018	Prospective RCT	3000	Shockmaster MP200 (Storz)	12 Hz	3–5 bar	8 sessions (2/week for 4 weeks).
Al Edwan et al. 2017	Prospective	2500	E-S.W.T Roland (Pagani, Italy)	3 Hz	0.25 mJ/mm^2^	4 weekly sessions.

### Indications, patient populations, and treatment parameters

Sample sizes of the included studies ranged from 40 participants [25,31] to 425 participants [37] (Table 2). These studies evaluated LiSWT in diverse patient populations across a range of clinical indications. Among those on ED, most studies focused on patients with vasculogenic ED [34,35,37], with subgroups including diabetic patients [25,28,33], PDE-5 inhibitor non-responders [35], and men with post-surgical ED following nerve-sparing radical cystoprostatectomy (NS-RCP) [29]. The severity of ED varied across studies, with several specifying inclusion of patients with mild to moderate dysfunction [25–27], while others included broader or unspecified severity ranges.

**Table 2.** LiSWT outcomes in erectile dysfunction or chronic pelvic pain syndrome.

Study author	Study population	Study group	Comparator group	Assessment tool	Follow-up duration	Summary of results
***Studies on erectile dysfunction***						
Abd El-Salam et al. 2024	ED patients (*n* = 150)	Naïve LiSWT group (*n* = 75)	Non-compliant PDE5i group (*n* = 75)	IIEF, EHS, PDU	6 weeks	*In naïve patients*: IIEF scores increased from 14.19 ± 2.54 to 22.23 ± 5.85; EHS scores increased from 2.27 ± 0.34 to 3.43 ± 0.64. *In the non-compliant group*: IIEF scores increased from 13.72 ± 2.79 to 21.82 ± 5.72; EHS scores increased from 2.18 ± 0.34 to 3.58 ± 0.58. No statistically significant difference between the studied groups in both outcome measures [*p* > 0.05]. No statistically significant difference between groups with regards to doppler findings.
Adeldaeim et al. 2020	Vasculogenic ED (*n* = 425)	–	–	SHIM	6 months	51.8% reported satisfactory intercourse; response sustained in 76.3% at 30 months; several clinical factors influenced success (Age, diabetes, hypertension, smoking, obesity, hyperlipidemia, pretreatment SHIM score, and the duration of ED).
Ismail et al. 2022	Diabetic ED patients on PDE5i (*n* = 40)	LiSWT (*n* = 20)	Sham (*n* = 20)	IIEF-5	1, 3, and 6 months	Pre-treatment IIEF-5 score in treatment group was 15.6 ± 0.99, and in Sham group 16 ± 0.79. IIEF-5 scores *for treatment group* were significantly increased at 1 month were 19.3 ± 1.4% change 24%), at 3 months 18.85 ± 1.56% change 21%), and 6 months 18.35 ± 1.3% change 15.6%) (*p* < 0.001 for all). IIEF-5 scores *for Sham group* were significantly increased at 1 month were 17.25 ± 1.01% change 8%), at 3 months 17.75 ± 0.9% change 11%), and 6 months 17.85 ± 0.8% change 11.5%) (*p* < 0.001 for all). After 1- and 3-month follow-up, there was a significant difference between the active and sham groups in favor of the active Li-ESWT group (p-value of 0.0001 and 0.01, respectively), but there was no significant difference between the groups at 6-month follow-up.
Feghali 2023	Vasculogenic ED (*n* = 85)	Based on ICI response	–	ICI response	NA	33 patients showed a robust response to the 10mcg ICI and were treated with four LISWT sessions. 52 patients exhibited a partial response to the 10mcg ICI, necessitating six LISWT sessions. 78.6% (26 out of 33) of the patients who initially responded well to 10 mcg ICI had a consistent response to 8 mcg ICI; 63.6% (33 out of 52) of those with a partial initial response to 10 mcg ICI demonstrated an improved reaction to the same dosage post-treatment.
Musa et al. 2020	Vascular ED refractory to PDE5i (*n* = 52)	–	–	IIEF, EHS	3, 6, 12, and 18 months	At the 18-month follow-up, 33 patients (63.5%) were able to achieve an erection sufficient for penetration with or without PDE5i (22 were maintained on oral PDE5i). The remaining 19 patients (36.5%) had a poor response to Li-SWT and oral PDE5i. The initial response showed some decline in 50% of the initial responders. Younger men (aged < 45 years), short ED duration ( < 2 years), and moderate ED severity responded better to Li- SWT. There were no adverse side-effects.
Salama et al. 2023	DM + ED (*n* = 45)	LiSWT, PE, or both	–	IIEF-5	4 and 12 weeks	The mean IIEF-5 scores significantly improved in all groups at the 4-week follow-up without intergroup differences. (group1: 12.13 ± 1.24 vs 15.6 ± 1.3; group2: 12.53 ± 1.3 vs 14.2 ± 1.01, group3: 12.26 ± 1.03 vs 16.33 ± 1.23) At the 12-week follow-up, the mean IIEF-5 improvement and durability were significantly higher among patients in combined groups. (group1: 13.66 ± 1.17, group2: 15.4 ± 1.18, group3: 18.2 ± 0.86.
Shakban et al. 2023	Vasculogenic mild-moderate ED (*n* = 60)	LiSWT (*n* = 30)	Sildenafil (*n* = 30)	EHS, IIEF, SEAR	1 and 3 months	Significant differences in EHS, IIEF-5, and SEAR scores at the treatment of the first and third months of in both groups when compared with level of baseline. *Group A (LiSWT)*– EHS: 1.77 ± 0.63 vs 2.43 ± 0.57 vs 3.10 ± 0.80, *p* < 0.01; IIEF-5: 15.80 ± 2.11vs 19.27 ± 1.31 vs 21.77 ± 2.47, *p* < 0.01; SEAR: 37.0 ± 5.50 vs 43.53 ± 2.19 vs 48.57 ± 3.77, *p* < 0.01. *Group B (Sildenafil)* – EHS: 1.90 ± 0.66 vs 2.70 ± 0.65 vs 3.23 ± 0.63, *p* < 0.01; IIEF-5: 15.37 ± 2.37 vs 23.57 ± 1.57 vs 21.70 ± 1.64, *p* < 0.01; SEAR: 36.37 ± 6.99 vs 51.57 ± 5.46 vs 46.93 ± 4.46, *p* < 0.01. No significant differences in the outcome measures were observed between group A and Group B.
Shendy et al. 2020	ED + diabetic polyneuropathy (*n* = 42)	LiSWT + pelvic therapy (*n* = 21)	Sham + pelvic therapy (*n* = 21)	IIEF-5, Doppler	3 months	After treatment, PSV of the right and left cavernous arteries increased significantly in the two groups. However, the post-treatment PSV was significantly higher in the LiSWT group compared to the sham group (*p* < .001, for both arteries). IIEF-5 increased significantly in the LiSWT group (12.75 ± 3.21 vs 17.50 ± 2.72, *p* < .001), but not in the control group (12.75 ± 2.61 vs 13.40 ± 2.85, *p* = 0.194) In the LiSWL group, 15 (71%) patients achieved erection sufficient for penetrative intercourse versus 2(9.5%) patients in the control group (*p* < 0.001)
Zanaty et al. 2022	Men with ED (*n* = 51)	LiSWT (*n* = 25)	Tadalafil 20 mg (*n* = 26)	IIEF-5, EHS, SEAR	6 and 12 weeks	The baseline IIEF-5 scores in the LiSWT and Tadalafil groups were 11.16 ± 4.2 and 10.08 ± 3.8 (*p* > 0.34). After the 6 and 12-week follow-ups, both groups showed significant improvement when comparing the baseline values to the follow-up variables for all IIEF-5, EHS, and SEAR (*p* < 0.05). LiSWT group: IIEF-5 - 11.16 ± 4.239 vs 16.36 ± 3.81 vs 17.64 ± 4.009. EHS − 1.64 ± 0.490 vs 3.1 ± 0.57 vs 3.2 ± 0.764. SEAR − 23.48 ± 2.57 vs 54.4 ± 4.25 vs 57.36 ± 3.79. Tadalafil group: IIEF-5 - 10.08 ± 3.818 vs 17.52 ± 2.75 vs 15.72 ± 3.635. EHS − 1.48 ± 0.586 vs 3.2 ± 0.46 vs 3.1 ± 0.690. SEAR − 23.84 ± 2.58 vs 57 ± 3.5 vs 54.6 ± 4.22. SEAR scores were significantly higher in the tadalafil group at 6 weeks compared with LiSWT group. No other between group differences were significant. There was a notable statistical difference between the two groups regarding the side effects, as the shockwave group was with mild side effects (8%), while the Tadalafil group (44%) of patients had side effects (*p* < 0.05)
Zewin et al. 2018	Post NS-RCP patients (*n* = 152)	LiSWT (*n* = 42)	PDE5i (*n* = 43), Control (*n* = 43)	IIEF-EF, EHS, PDU	1, 3, 6 and 9 months	At 1 month, all patients of the three groups had an insufficient erection for vaginal penetration (EHS ≤2), with the decrease of preoperative IIEF-EF mean score from 27.9 ± 0.7 to 6.9 ± 0.7 and EHS mean score from 3.9 ± 0.3 to 1.9 ± 0.3. In the three groups, statistically significant increase in total IIEF score and EHS from 1 to 3, 6, and 9 months (*p* < 0.001 for all check points). Potency recovery rates at 3 months were 45.2%, 55.8%, and 34.9% in LI-ESWT, PDE5i, and control groups, respectively, while potency recovery rates at 9 months were 76.2%, 79.1%, and 60.5% in LI-ESWT, PDE5i, and control group, respectively. No significant difference in potency recovery rates, EDV or PSV during the follow-up periods among the groups
***Studies on chronic pelvic pain syndrome***						
Khalafalla et al. 2023	CP/CPPS patients (*n* = 75)	–	–	NIH-CPSI	0, 12 and 26 weeks	Compared to pre-treatment scores, all patients exhibited improvements across all NIH – CPSI domains directly after completing LiSWT (week 0 post-treatment), with a mean difference improvement of 9.26 ± 5.7, 5.2 ± 3.4, 1.19 ± 2.18 and 2.88 ± 2.46 points in the total, pain, urinary symptoms, and QoL scores respectively. At 12 weeks after completing LiSWT, 80.9% of patients reported improvements, with mean difference improvement of 8.07 ± 7.56, 4.55 ± 4.6, 0.76 ± 2.48, 2.85 ± 2.78 in the total, pain, urinary symptoms, and QoL scores respectively. At 26 weeks after completing LiSWT, 82.4% of patients reported improvements, with mean difference improvement of 8.29 ± 7.7%, 4.92 ± 4.69, 0.75 ± 2.96, 2.5 ± 3.0 in total, pain, urinary symptoms, and QoL scores respectively.
Sakr et al. 2022	CPPS patients (*n* = 155)	LiSWT group (*n* = 78)	Placebo group (*n* = 77)	NIH-CPSI, IPSS, IIEF-5, VAS	1, 3, 6, and 12 months	A statistically significant improvement was noticed in the mean values of NIH-CPSI, VAS, and IIEF-5 of the treatment group patients from the baseline criteria over the follow-up period. There was a statistically significant difference between both groups regarding the mean values of NIH-CPSI, IPSS, VAS, and IIEF-5 throughout the period of follow-up. At the first visit of follow-up 1 month after LiSWT, 63 (82.8%) patients of the treatment group had ≥6 points decrease in the NIH-CPSI total score, while 13 (17.2%) patients did not. All the 63 patients who showed well response to LiSWT from the treatment group at the 1 month follow-up could maintain their response throughout follow-up period.
Salama and Abouelnaga 2018	CPPS patients (*n* = 40)	LiSWT (*n* = 20)	Control (*n* = 20)	NIH-CPSI	1 week after the initiation of the treatment (post-1), 4 weeks after the initiation of the treatment (post-2), and 8 weeks after the end of treatment (post-3)	In the treatment group, the means of all four domains of the NIH-CPSI score significantly decreased in post-1, post-2, and post-3 from the baseline (*p* < 0.05). Also, there was significant decrease in post-2 and post-3 as compared to post-1 (*p* < 0.05). However, no significant differences were found in all four domains between post-2 and post-3 (*p* > 0.05). In the control group, there was a significant decrease in all four domains of the NIH- CPSI score in post-1 as compared to the baseline (*p* < 0.05), but there was no significant difference in post-2 and post-3 as compared to the previous reading (*p* < 0.05).
Al Edwan et al. 2017	CPPS patients (*n* = 41)	–	–	VAS, NIH-CPSI, IPSS, QOL, IIEF	2 weeks, 6 months and 12 months	Significant improvement in all parameters, maintained at 6 and 12 months.

The duration of treatment and treatment frequency also varied. Most protocols involved delivering LiSWT twice weekly, over a span ranging from 3 to 6 weeks, sometimes followed by a treatment-free rest period and subsequent retreatment [25,35]. Shockwave protocols differed in energy flux density (ranging from 0.09 to 0.25 mJ/mm^2^), total number of shocks per session (from 1500 to 6000), and treatment site distribution. Devices used included focused systems (e.g. PiezoWave2, Duolith SD1 Ultra, Dornier Aries) and radial systems (e.g. Chattanooga Intelect RPW, Shockmaster MP200). While three studies explicitly excluded concurrent PDE5i use [26,27,29], the remaining studies either allowed continuation or did not specify their approach (Table 1).

Assessment of treatment response relied on validated tools such as the International Index of Erectile Function (IIEF or IIEF-5), Erection Hardness Score (EHS), Penile Duplex Ultrasound (PDU), Sexual Health Inventory for Men (SHIM), and Self-Esteem and Relationship Questionnaire (SEAR). For CP/CPPS, outcomes were assessed using the National Institutes of Health Chronic Prostatitis Symptom Index (NIH-CPSI), the International Prostate Symptom Score (IPSS), the Visual Analog Scale (VAS), and quality of life indices.

### Efficacy of LiSWT in erectile dysfunction

The included studies on ED demonstrated consistent benefits of LiSWT, albeit with some variations depending on comparator groups and patient characteristics (Table 2).

Two RCTs compared LiSWT to sham treatments. Ismail and Abd El-Azeim (2022) observed significant increases in IIEF-5 scores in both groups at 1, 3, and 6 months, with LiSWT showing greater efficacy at 1 and 3 months, although the differences were not sustained at 6 months [25]. Despite observing significant increases in PSV in both treatment and sham groups after 3 months of the intervention, Shendy et al. (2020) reported significant improvements in IIEF-5 scores only in the LiSWT group, with 71% of patients achieving erections sufficient for penetration compared to 9.5% in the sham group [28].

Three RCTs compared LiSWT to PDE-5 inhibitors (PDE-5is). Both Zanaty et al. (2022) and Shakban et al. (2023) found significant improvements in IIEF-5, EHS, and SEAR scores in both groups, with no significant differences between LiSWT and pharmacological treatment [26,27]. Zewin et al. (2018) assessed the effectiveness of LiSWT, PDE-5is, and a control group in patients who underwent NS-RCP. They reported significant improvements in IIEF-EF, EHS, and PDU scores across all groups at 3, 6, and 9 months post-treatment, with no significant differences between the groups [29]. Potency rates at 9 months were 76.2% for the LiSWT group, 79.1% for the PDE-5i group, and 60.5% for the control group, with no statistically significant differences among them [29].

Non-randomized comparative studies highlighted the utility of LiSWT in specific populations. Salama et al. (2023) found that combining LiSWT with physical exercise produced the greatest improvements in IIEF-5 scores among diabetic patients [33]. Abd El-Salam et al. (2024) reported similar improvements in IIEF and EHS scores among PDE-5i-naïve and non-compliant patients, with no significant differences between these groups [32].

Observational studies supported the efficacy of LiSWT, reporting improvements in erectile function with rates of satisfactory sexual intercourse ranging from 51.8% to 76.3% over follow-up periods up to 30 months [34,35,37].

### Efficacy of LiSWT in CP/CPPS

Four studies evaluated the impact of LiSWT in patients with CP/CPPS, including two RCTs [30,31] and two observational studies [36,38] (Table 2).

Salama and Abouelnaga (2018) observed significant reductions in NIH-CPSI scores in both treatment and sham groups after 1 week, with sustained improvements at 4 and 8 weeks seen exclusively in the treatment group [31]. Sakr et al. (2022) used multiple assessment tools, including NIH-CPSI, IPSS, and IIEF-5, and reported clinically meaningful improvements in 82.8% of patients in the treatment group, with benefits evident as early as 1 month and sustained for up to 12 months [30].

Khalafalla et al. (2023) reported significant reductions in pain and urinary symptoms (NIH-CPSI) in 80.9% and 82.4% of patients at 12 and 26 weeks, respectively [36]. Al Edwan et al. (2017) demonstrated statistically significant improvements across all measures, including NIH-CPSI and IPSS, with the maximum improvements observed earlier (2 weeks) for pain and QoL domains compared with those observed for urinary symptoms (6 months) [38].

### Predictors of success, safety, and tolerability

Several studies identified factors influencing the efficacy of LiSWT. Younger patients (<45 years) and those with shorter ED duration (<2 years) achieved better outcomes [35]. Patients with milder ED also responded more favorably [34].

Across all studies, LiSWT was well-tolerated with minimal adverse effects. Only 8% of patients in the LiSWT group reported mild side effects, compared to 44% in the Tadalafil group [27].

### Quality assessment of included studies

The quality of the included studies was evaluated using standardized quality assessment tools. RCTs were assessed using the Cochrane Risk of Bias Tool, while observational and non-randomized comparative studies were evaluated using the Newcastle-Ottawa Scale (NOS). Overall, the quality of the studies ranged from moderate to high (Tables 3 and 4).

**Table 3.** Quality assessment of randomized controlled trials (Cochrane Risk of Bias Tool).

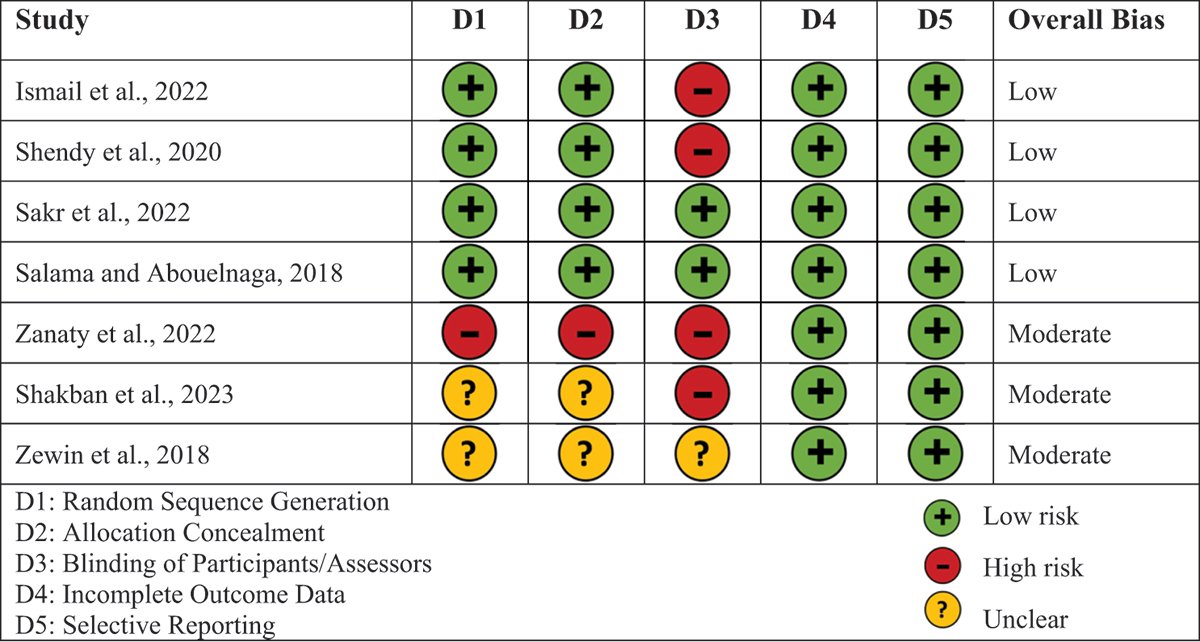

**Table 4.** Quality Assessment of Observational Studies (Newcastle-Ottawa Scale).

Study	Selection (Max ★★★★)	Comparability (Max ★★)	Outcome (Max ★★★)	Total (Max 9)	Overall Quality
Feghali, 2023	★★★★	★★	★★★	9	High
Musa et al., 2020	★★★★	★★	★★★	9	High
Khalafalla et al., 2023	★★★	★	★★★	7	Moderate
Adeldaeim et al., 2020	★★★★	★★	★★★	9	High
Al Edwan et al., 2017	★★★	★	★★	6	Moderate
Abd El-Salam et al. 2020	★★★	-	★★	5	Low
Salama et al. 2023	★★★	★	★★	6	Moderate

Among the seven RCTs, four demonstrated a low risk of bias across domains, including random sequence generation, allocation concealment, and outcome reporting [25,28,30,31]. However, three RCTs showed a high risk of bias in domains such as blinding of participants and assessors, primarily due to logistical challenges in implementing double-blinding [26,27,29].

For non-randomized studies, NOS scores ranged from 5 to 8, indicating moderate to high quality. Common strengths included well-defined cohorts, adequate follow-up, and adjustment for confounders. However, some studies lacked clarity in participant selection criteria and comparability between groups, which may have introduced selection bias [36,38].

## Discussion

This systematic review evaluated the efficacy and safety of LiSWT in managing ED and CP/CPPS within the Middle Eastern population. The included studies consistently reported significant improvements in erectile function and reductions in CP/CPPS-related pain and urinary symptoms, underscoring LiSWT’s potential as a non-invasive therapeutic option. However, the findings also revealed notable variability in treatment protocols together with limited long-term efficacy.

### Effect on ED

The two RCTs included in this review comparing LiSWT to sham treatment demonstrated significant short-term improvements in erectile function [25,28]. However, no sham-controlled RCTs from the Middle East have assessed the effects of LiSWT beyond six months. While both studies observed significant benefits at three months, these effects were not sustained at six months [25,28]. This aligns with global findings reported by Brunckhorst et al. (2019), who conducted a systematic review of eleven studies involving 799 patients [39]. Of the five studies evaluating erectile function at 12 months, two reported a plateau in results, while three showed a deterioration in IIEF-EF scores (ranging from −2 to 0.1) between six and twelve months.

It is also noteworthy that the two studies included in this review observed significant improvements in IIEF-5 or PSV scores within the sham groups, suggesting a potential placebo effect [25,28]. This phenomenon has also been reported by others; For instance, Fojecki et al. (2017) found no significant differences between groups but noted that success rates based on IIEF-EF scores were 38.3% in the sham group and 37.9% in the LiSWT group (odds ratio = 0.95, 95% CI = 0.45–2.02, *p* = .902). Similarly, success rates based on EHS scores were 6.7% in the sham group and 3.5% in the LiSWT group (odds ratio = 0.44, 95% CI = 0.08–2.61, *p* = .369) [40].

Two RCTs have compared LiSWT to PDE-5is. Zanaty et al. (2022) and Shakban et al. (2023) reported no significant differences in IIEF-5 and EHS scores between the two treatments, indicating comparable efficacy [26,27]. Several studies have explored the combination of LiSWT and PDE-5is, suggesting that LiSWT may have an additive effect on erectile function, particularly in patients with vasculogenic ED or those unresponsive to PDE-5i*s* [41,42]. To the best of our knowledge, only one study directly compared LiSWT to on-demand sildenafil in 78 participants (46 in the LiSWT group and 32 in the sildenafil group) [43]. Based on the IIEF score, the proportion of improvement, defined by minimal clinically important difference (MCID) criteria, was 52.2% in the LiSWT group and 59.4% in the sildenafil group (*p* > 0.05). Similarly, improvements in EHS and SEAR scores were comparable between the two groups (*p* > 0.05 at baseline and after three months). These findings, combined with the results of the two studies included in this review, suggest that LiSWT may be a viable alternative for men who are unable to take oral medications due to clinical contraindications or personal preferences.

Zewin et al. (2018) specifically evaluated LiSWT in patients following NS-RCP and reported no significant differences in potency rates between LiSWT (76.2%), PDE-5is (79.1%), and a control group (60.5%). These findings are consistent with the systematic review by Sighinolfi et al., which examined the role of LiSWT in managing ED after radical prostatectomy. The review included nine studies published between 2015 and 2022, three of which were RCTs. Unlike the study by Zewin et al., most of the studies in Sighinolfi et al.’s review compared the combination of LiSWT and PDE-5is with PDE-5is alone. The review found a modest but statistically significant improvement in erectile function with combination therapy compared to PDE-5is alone, albeit with a lack of standardization and considerable heterogeneity in the LiSWT treatment protocols used.

The inclusion of a control group in Zewin et al.’s study provides valuable insights by demonstrating the role of natural recovery in post-surgical ED and offers sound comparisons with interventions used for penile rehabilitation. Collectively, these findings suggest that LiSWT serves better as an adjunctive therapy rather than a definitive standalone treatment, underscoring the need for further RCTs with larger sample sizes to firmly establish its efficacy.

Two non-randomized comparative studies explored LiSWT in specific patient groups. Salama et al. (2023) demonstrated that combining LiSWT with physical exercise in diabetic patients led to significantly greater IIEF-5 improvements than LiSWT alone [33]. This suggests that multimodal interventions addressing both vascular and lifestyle factors may enhance outcomes in populations with severe baseline vascular compromise. A systematic review by Silva et al. (2017) further supports the benefits of regular physical activity in improving erectile function, reinforcing the rationale for combination approaches [44].

Abd El-Salam et al. (2024) evaluated LiSWT in PDE-5i-naïve and non-compliant patients, finding comparable improvements in IIEF-5 and EHS scores in both groups. These results highlight LiSWT’s utility as a primary treatment for patients unable or unwilling to use pharmacological therapies. Similarly, Bechara et al. (2016) evaluated the long-term efficacy and safety of LiSWT in 50 patients with ED unresponsive to PDE5is [45]. Throughout the study, patients continued their regular PDE5i regimens. At the 12-month follow-up, 60% of participants exhibited significant improvements in erectile function, as measured by the IIEF-EF, SEP2 and SEP3, EHS, and GAQ scores. These findings reinforce LiSWT’s role as a viable option for diverse patient populations, including those unresponsive to PDE-5is.

### Effect on CP/CPPS

Our review highlights the significant benefits of LiSWT for CP/CPPS and the improvements, assessed using tools such as NIH-CPSI, IPSS, and IIEF-5, were sustained for up to 12 months [30,31,36,38]. Similar findings have been reported in global systematic reviews. Kong et al. analyzed 12 RCTs (651 patients) and found significant reductions in NIH-CPSI pain scores at treatment completion and at 1, 4, 12, and 24 weeks post-treatment compared to controls and baseline [46]. The total NIH-CPSI and QoL scores also showed sustained improvements. However, urinary domain improvements were only significant at treatment completion and at 1 and 12 weeks, while the IPSS scores, were significantly reduced at 1, 4, and 12 weeks post-treatment.

Another systemic review demonstrated that the effects of LiSWT appear to be more pronounced in alleviating pain and overall QoL than in addressing associated urinary symptoms. Mykoniatis et al. (2021) analyzed five RCTs and found that LiSWT primarily alleviates pain and improves QoL, with limited effects on urinary symptoms [10]. While NIH-CPSI urinary scores improved immediately post-treatment and IPSS scores showed benefits up to three months, LiSWT had no significant impact on PVR or Qmax, suggesting it may not directly address the underlying voiding dysfunction in CP/CPPS patients.

### Protocol variability

The review identified significant variability in treatment protocols, including energy densities ranging from 0.09 to 0.25 mJ/mm^2^, shocks per session from 1500 to 6000, and total sessions ranging from 4 to 12. Sessions were delivered once or twice weekly, with follow-up durations inconsistently reported. These findings align with broader literature emphasizing the need for standardized protocols. Systematic reviews by Brunckhorst et al. (2019) and Mykoniatis et al. (2023) similarly highlighted protocol heterogeneity, which complicates cross-study comparisons and limits generalizability [39,42]. This variability in treatment protocols poses a challenge in determining the optimal LiSWT regimen for clinical use, as differences in energy density, frequency, and duration of treatment may influence therapeutic outcomes. Without standardized protocols, it becomes difficult to compare study results, draw definitive conclusions about efficacy, and establish clear clinical guidelines for patient selection and treatment optimization.

### Limitations

This review has several limitations. Variability in LiSWT protocols among included studies, as stated above, hinders direct comparisons and meta-analyses. Because many studies involved small sample sizes, were conducted in private-sector environments that may introduce bias, and had short follow-up periods, the strength of the long-term efficacy conclusions is reduced. Additionally, the regional focus on the Middle East may limit the applicability of findings to other populations. Finally, the lack of consistent reporting on blinding and allocation concealment in some studies introduces a risk of bias.

## Conclusion

LiSWT has emerged as a promising non-invasive treatment option for ED and chronic CP/CPPS. Our review highlights its efficacy in improving erectile function, reducing CP/CPPS symptoms, and enhancing quality of life in the Middle Eastern population. However, the findings underscore notable limitations, including variability in treatment protocols, lack of long-term efficacy data, and challenges in standardizing clinical applications.

While LiSWT demonstrates potential as a primary or adjunctive therapy, especially for patients unable or unwilling to use pharmacological treatments, its role remains investigational. Addressing protocol heterogeneity through well-designed, large-scale RCTs and establishing standardized guidelines are essential to optimize its clinical utility. Until such evidence is available, clinicians should carefully consider patient selection and individual circumstances when incorporating LiSWT into practice.

## Supplementary Material

Supplemental Material
