# Supplementary material for: Low-intensity shockwave therapy in men’s health: Insights from a systematic review on erectile dysfunction and CP/CPPS in the Middle East
Source: Arab J Urol. 2026 Jan 5;24(4):277–88. doi: 10.1080/20905998.2025.2611547 (PMC13637759; doi:10.1080/20905998.2025.2611547)
Supplement: Supplementary file 1 [file TAJU_A_2611547_SM3739.docx]

**Supplementary table 1. Keywords used in the literature search**

Shock wave therapy in the Middle East region:

("LiSWT"[All Fields] OR (("shock"[MeSH Terms] OR "shock"[All Fields] OR "shocked"[All Fields] OR "shocking"[All Fields] OR "shocks"[All Fields]) AND "wave"[All Fields]) OR "ESWT"[All Fields] OR "ESWL"[All Fields] OR ("low"[All Fields] AND ("intense"[All Fields] OR "intensely"[All Fields] OR "intensities"[All Fields] OR "intensity"[All Fields] OR "intensively"[All Fields]) AND ("shock"[MeSH Terms] OR "shock"[All Fields] OR "shocked"[All Fields] OR "shocking"[All Fields] OR "shocks"[All Fields]))) AND ("lebanon"[Affiliation] OR "syria"[Affiliation] OR "jordan"[Affiliation] OR "palestine"[Affiliation] OR "iraq"[Affiliation] OR "saudi arabia"[Affiliation] OR "yemen"[Affiliation] OR "oman"[Affiliation] OR "united arab emirates"[Affiliation] OR "bahrain"[Affiliation] OR "kuwait"[Affiliation] OR "qatar"[Affiliation] OR "egypt"[Affiliation] OR "tunisia"[Affiliation] OR "lybia"[Affiliation] OR "algeria"[Affiliation] OR "morocco"[Affiliation] OR "Djibouti"[Affiliation] OR "somalia"[Affiliation] OR "mauritania"[Affiliation] OR "comoros"[Affiliation])

Erectile dysfunction:

("erectile dysfunction"[MeSH Terms] OR ("erectile"[All Fields] AND "dysfunction"[All Fields]) OR "erectile dysfunction"[All Fields] OR ("erections"[All Fields] OR "erective"[All Fields] OR "penile erection"[MeSH Terms] OR ("penile"[All Fields] AND "erection"[All Fields]) OR "penile erection"[All Fields] OR "erection"[All Fields]) OR ("education"[MeSH Subheading] OR "education"[All Fields] OR "ed"[All Fields]) OR ("penis"[MeSH Terms] OR "penis"[All Fields]) OR "erectil*"[All Fields])

Peyronie’s disease:

("peyronie"[All Fields] OR "peyronie s"[All Fields] OR "peyronies"[All Fields] OR ("pharmacology"[MeSH Subheading] OR "pharmacology"[All Fields] OR "pd"[All Fields]) OR ("curvature"[All Fields] OR "curvatures"[All Fields]) OR ("penile induration"[MeSH Terms] OR ("penile"[All Fields] AND "induration"[All Fields]) OR "penile induration"[All Fields] OR ("penile"[All Fields] AND "plaque"[All Fields]) OR "penile plaque"[All Fields]))

Chronic pelvic pain:

("chronic"[All Fields] OR "chronical"[All Fields] OR "chronically"[All Fields] OR "chronicities"[All Fields] OR "chronicity"[All Fields] OR "chronicization"[All Fields] OR "chronics"[All Fields]) AND ("prostat"[All Fields] OR "prostate"[MeSH Terms] OR "prostate"[All Fields] OR "prostates"[All Fields] OR "prostatic"[All Fields] OR "prostatism"[MeSH Terms] OR "prostatism"[All Fields] OR "prostatitis"[MeSH Terms] OR "prostatitis"[All Fields])) OR (("chronic"[All Fields] OR "chronical"[All Fields] OR "chronically"[All Fields] OR "chronicities"[All Fields] OR "chronicity"[All Fields] OR "chronicization"[All Fields] OR "chronics"[All Fields]) AND ("pelvic pain"[MeSH Terms] OR ("pelvic"[All Fields] AND "pain"[All Fields]) OR "pelvic pain"[All Fields])) OR ("cogn process"[Journal] OR "CP"[All Fields]) OR "CPPS"[All Fields] OR "cp cpps"[All Fields] OR ("pelvic pain"[MeSH Terms] OR ("pelvic"[All Fields] AND "pain"[All Fields]) OR "pelvic pain"[All Fields]) OR ("pelvi"[All Fields] OR "pelvis"[MeSH Terms] OR "pelvis"[All Fields]) OR ("pain"[MeSH Terms] OR "pain"[All Fields]))
